# Phenotype, Function, and Mobilization of 6-Sulfo LacNAc-Expressing Monocytes in Atopic Dermatitis

**DOI:** 10.3389/fimmu.2018.01352

**Published:** 2018-06-21

**Authors:** Wojciech Baran, Stephanie Oehrl, Fareed Ahmad, Thomas Döbel, Christina Alt, Angelika Buske-Kirschbaum, Marc Schmitz, Knut Schäkel

**Affiliations:** ^1^Department of Dermatology, Venerology and Allergology, Wroclaw Medical University, Wroclaw, Poland; ^2^Department of Dermatology, Medical Faculty, University of Heidelberg, Heidelberg, Germany; ^3^Department of Biopsycology, Technical University of Dresden, Dresden, Germany; ^4^Institute of Immunology, Medical Faculty, Technical University of Dresden, Dresden, Germany; ^5^National Center for Tumor Diseases, University Hospital Carl Gustav Carus, Technical University of Dresden, Dresden, Germany; ^6^German Cancer Consortium (DKTK), Dresden, Germany; ^7^German Cancer Research Center (DKFZ), Heidelberg, Germany; ^8^Center for Regenerative Therapies Dresden (CRTD), Medical Faculty, Technical University of Dresden, Dresden, Germany

**Keywords:** atopic dermatitis, dendritic cells, 6-sulfo LacNAc-expressing monocytes, immunology, inflammation, IL-12, CD86

## Abstract

Mononuclear phagocytes (MPs) are important immune regulatory cells in atopic dermatitis (AD). We previously identified 6-sulfo LacNAc-expressing monocytes (slanMo) as TNF-α- and IL-23-producing cells in psoriatic skin lesions and as inducers of IFN-γ-, IL-17-, and IL-22-producing T cells. These cytokines are also upregulated in AD and normalize with treatment, as recently shown for dupilumab-treated patients. We here asked for the role of slanMo in AD. Increased numbers of slanMo were found in AD skin lesions. In difference to other MPs in AD, slanMo lacked expression of FcɛRI, CD1a, CD14, and CD163. slanMo from blood of patients with AD expressed increased levels of CD86 and produced IL-12 and TNF-α at higher amounts than CD14^+^ monocytes and myeloid dendritic cells. While CD14^+^ monocytes from patients with AD revealed a reduced IL-12 production, we observed no difference in the cytokine production comparing slanMo in AD and healthy controls. Interestingly, experimentally induced mental stress, a common trigger of flares in patients with AD, rapidly mobilized slanMo which retained their high TNF-α-producing capacity. This study identifies slanMo as a distinct population of inflammatory cells in skin lesions and as proinflammatory blood cells in patients with AD. slanMo may, therefore, represent a potent future target for treatment of AD.

## Introduction

Atopic dermatitis (AD) is the eczematous skin manifestation of atopy affecting 15–25% of children and 1–3% of adults (1). The pathogenesis of AD is multifactorial. According to current studies, the most important causes for the immune imbalance leading to skin inflammation in AD are skin barrier defects and the patients’ immunogenetic background (2–4). In addition, environmental factors such as exposure to allergens, microbial products, and psychosocial stress are relevant for disease manifestation (3, 5). Dendritic cells (DCs), monocytes, and macrophages, collectively called mononuclear phagocytes (MPs), are heterogeneous in their phenotype, function, and origin and play fundamental roles in regulating innate and adaptive immune responses in skin (6). Unraveling the phenotypic and functional specialization of individual MP subsets within the skin compartment will enhance our understanding of how in AD pathogenic T cell responses against allergens and autoantigens as well as the quality of these responses—Th2, Th1, Th22, and Th17—are promoted and antagonized.

In AD skin lesions, inflammatory dendritic epidermal cells (IDECs) (CD1a^+^, Lagerin^−^, FceRI^+^) are thought to enhance local inflammation and increase eczema severity by their proinflammatory capacity (7, 8). It was shown that these antigen-presenting cells in AD can take up antigens *via* IgE and FcɛRI to increase stimulation of allergen-specific T cells. In addition, an increased number of myeloid-derived suppressor cells (MDSCs) were recently described in AD skin lesions. MDSCs were induced by TLR2–TLR6 ligands and can cause consecutive immune suppression which may lead to defective local antimicrobial immune defense (9).

The changes within the MP system in AD are complex. In order to value better the distinct facets of AD, recent studies compared AD side by side with psoriasis (10). In AD, higher frequencies of dermal MPs expressing CD1a, CD11c, CD206, CD1b/c, and DC-SIGN were identified. In addition, the authors found an increased frequency of plasmacytoid DCs (pDCs) in AD compared to psoriasis (11). This latter finding was previously also demonstrated by Stary et al. (12). pDCs in AD produce CCL22 which supports the chemotaxis of Th2 T cells. IDECs initially described in the epidermis were also found in the dermis in patients with AD and to a lesser extend also in psoriasis (10). This systematic analysis, however, did not allow to specifically address the presence of 6-sulfo LacNAc (slan)-expressing cells, cells which were not identified using the markers CD1a, BDCA2, or CD1c, but which express CD11c (13).

We previously identified slan as an O-linked carbohydrate expressed on PSGL-1 and as a marker for a specific CD16^+^ myeloid cell population. Studies with slan^+^ cells from blood revealed their DC-like function with high level production of IL-12, IL-23, IL-1β, and TNF-α, the capacity to stimulate naïveT cells and to program Th1/Th17 cells. Others found slan^+^ cells isolated from human tonsils to reveal an unequivocal terminal differentiation to DCs. We identified increased numbers of slan^+^ cells in the dermis of psoriatic lesions where they produce IL-23, TNF-α, and iNOS and thereby representing inflammatory dermal DCs (TIP-DCs) (14, 15). Subsequently, a role for slan^+^ cells was found in lupus skin lesions as well as in several other organs or diseases such as in multiple sclerosis, Crohn’s disease, rheumatoid arthritis, or HIV (16–20). Although slan^+^ cells can clearly function as DCs, they may also differentiate into macrophages, as demonstrated in renal cell carcinoma (17, 19).

Dendritic cells, monocytes, and macrophages are now classified based on their identified precursor cell. For slan^+^ cells, transcriptomic studies collectively demonstrated a gene signature shared with the family of monocytes rather than *bona fide* DCs (21). We therefore, propose to call slan^+^ cells slan monocytes [6-sulfo LacNAc-expressing monocyte (slanMo)] instead of slan^+^ DCs. Monocytes are subdivided according to their gradual differences in CD14 versus CD16 expression: classical CD14^++^CD16^−^, intermediate CD14^+^CD16^+^, and non-classical CD14^−^CD16^++^ monocytes (22). slan^+^ cells fall into the category of CD16^+^ LIN^−^ HLA-DR^+^ leukocytes (non-classical CD14^−^CD16^++^ monocytes). Roughly half of the non-classical monocytes stain positive for slan. The population of human non-classical monocytes is regarded equivalent to mouse patrolling monocytes (23).

To increase our understanding of the immunopathogenesis of AD, we investigated in this study the presence, phenotype, and function of slanMo in skin and blood of patients with AD. We identified slanMo as a frequent, proinflammatory, and phenotypically distinct cell population in AD. Our study, therefore, provides an important new facet to the cellular immunopathogenesis of AD with a potential for translation into a slan-targeted therapy.

## Materials and Methods

### Patient Characteristics

The studies were approved by the ethics commission at medical faculties of Heidelberg (S033/13, S03/2013, S305/2010, S306/2010) and Dresden (2602/2007) in accordance with the declaration of Helsinki. A written informed consent was obtained from all participants of the studies.

#### Patients Included for Maturation Marker Expression/Cytokine Production

Blood was collected from seven patients with AD (mean age 23.08 ± 1.98) who did not receive systemic therapy for >4 weeks and topical steroids or calcineurin inhibitors for >14 days. Type I hypersensitivity was confirmed by a positive prick test to home dust mites (HDM) and HDM-specific IgE.

Patients included for immunohistochemistry and immunofluorescence analysis: skin biopsies (6 mm punch biopsy) were collected from 10 patients with atopic dermatitis (4 females and 6 males, mean age 53.2 ± 18.4 years). The patients did not receive systemic therapy for >4 weeks and topical steroids or calcineurin inhibitors for >14 days. Skin samples were also taken from 10 healthy volunteers (5 females and 5 males, mean age 42.2 ± 17.3 years) with no personal and family history of atopic dermatitis (control group).

#### Patients Included in Tier Social Stress Test (TSST)

Blood was collected from 16 patients with AD. All patients had elevated IgE levels (range 150–31,040 IU/ml, mean 9,314 ± 10,508 IU/ml) and an exacerbation of chronic AD (Scoring Atopic Dermatitis Index between 20 and 70). 10 females (mean age 31.50 ± 8.35) and 6 males (mean age 37.50 ± 14.36) were included.

### Immunohistochemistry and Immunofluorescence Analysis

Paraffin-embedded tissues were cut into 2–5 µm sections and rehydrated. Antigen retrieval was performed by boiling for 20 min in sodium citrate (Target retrieval solution, Dako; Hamburg, Germany). For immunohistochemistry application of the blocking agent (Dual endogenous enzyme block, Dako) the primary antibody DD2 (IgM, 10 μg/ml hybridoma supernatant, generated in our lab) was applied, followed by the goat anti-mouse IgM-biotin (Southern Biotech; Melbourne, VIC, Australia) and the Dako Real Detection System (Dako). Sections were counterstained with Mayer’s hematoxylin (Sigma-Aldrich, Hamburg, Germany).

The primary antibodies for immunofluorescent staining to evaluate the phenotype slanMo in tissue were: anti-slan (clone DD2), anti-HLA-DR (mouse IgG1, TAL.1B5, Dako), anti-CD1a (mouse IgG1, clone: O10, Beckmann Coulter, Krefeld, Germany), anti-CD14 (mouse IgG2a, clone: 7, Thermo Fisher Scientific; Darmstadt, Germany), anti-CD163 (mouse IgG1, clone: 10D6, Leica, Wetzlar, Germany), and anti-FcεRIα (mouse IgG2b, clone 9E1, Acris; Herford, Germany). As secondary antibodies Alexa Fluor 488 conjugated anti-mouse IgG-specific goat F(ab′)2 (Invitrogen, Karlsruhe, Germany) and biotinylated anti-mouse IgM-specific donkey F(ab′)2 fragments (Jackson ImmunoResearch, Suffolk, United Kingdom) were used followed by avidin D sulforhodamine 101 (Vector Laboratories, Burlingame, CA, USA). Cell nuclei were counterstained with DAPI (BioLegend, San Diego, CA, USA). Sections were studied with a Zeiss Axio Imager A1 microscope. For quantification of cells in histologic sections, up to six pictures of each section were taken, and the dermal area in each picture was determined by Image J software (Image Processing and Analysis in Java, freeware, http://rsbweb.nih.gov/ij/). Positively stained cells were counted, and the density was calculated per square millimeter.

### Cell Culture

Peripheral blood mononuclear cells (PBMCs) were prepared by Ficoll gradient centrifugation (Biochrom AG, Berlin, Germany) and resuspended in RPMI medium supplemented with 2 mM l-glutamine, 1% non-essential amino acids, 100 U/mL penicillin, 100 µg/ml streptomycin, and 10% heat-inactivated pooled human AB serum (CC Pro, Oberdorla, Germany) and stimulated at 6 h of culture with 100 ng/ml LPS (*Escherichia coli* 026:B6; Sigma-Aldrich) supplemented with 1 µg/ml brefeldin A (Sigma-Aldrich) to inhibit cytokine secretion and cultured for a total of 24 h (37°C at 5% CO_2_). For detection of TNF-α in slanMo before and after participation in the stress test, blood was taken, PBMCs were stimulated with LPS for 6 h, and subsequently stained for TNF-α.

### Frequency and Phenotype of slanMo

Freshly isolated or 24-h cultured PBMCs were cell surface stained and analyzed by flow cytometry [FACS Canto, Becton Dickinson (BD), Heidelberg, Germany]. The following mAbs were used at optimal dilutions: anti-slan (M-DC8 hybridoma supernatant raised in our laboratory) followed by anti-μ-PE or -FITC [goat F(ab′)2 anti-mouse IgM, Beckmann Coulter], anti-CD83, anti-CD86, and anti-HLA-DR (all from BD Pharmingen) and appropriate isotype-matched control mAbs.

### TNF-α and IL-12 Production

For intracytoplasmic cytokine staining PBMCs first underwent cell surface staining to identify slanMo, CD11c^+^ cDCs, pDCs, and monocytes. Cells were then fixed in fresh ice-cold 4% paraformaldehyde, and permeabilized using 0.1% saponin in PBS containing 1% FCS. For intracellular cytokine staining an anti-TNF-α mAb (clone MAb11), an anti-IL-12/23p40 mAb (clone 11.5, both BD), and respective isotype controls were used.

### Mental Stress Test

All subjects were exposed to the “Trier Social Stress Test” (TSST), which has been described and evaluated elsewhere (24). Briefly, the TSST is a standardized laboratory stressor that mainly consists of a free speech (job interview) and mental arithmetic tasks (serial subtraction) in a role-playing approach in front of an audience. Blood was drawn from an intravenous catheter at the indicated time points (Figure 5) and PBMCs prepared and stimulated as described above.

### Evaluation and Statistical Analysis

**Figure 1:**
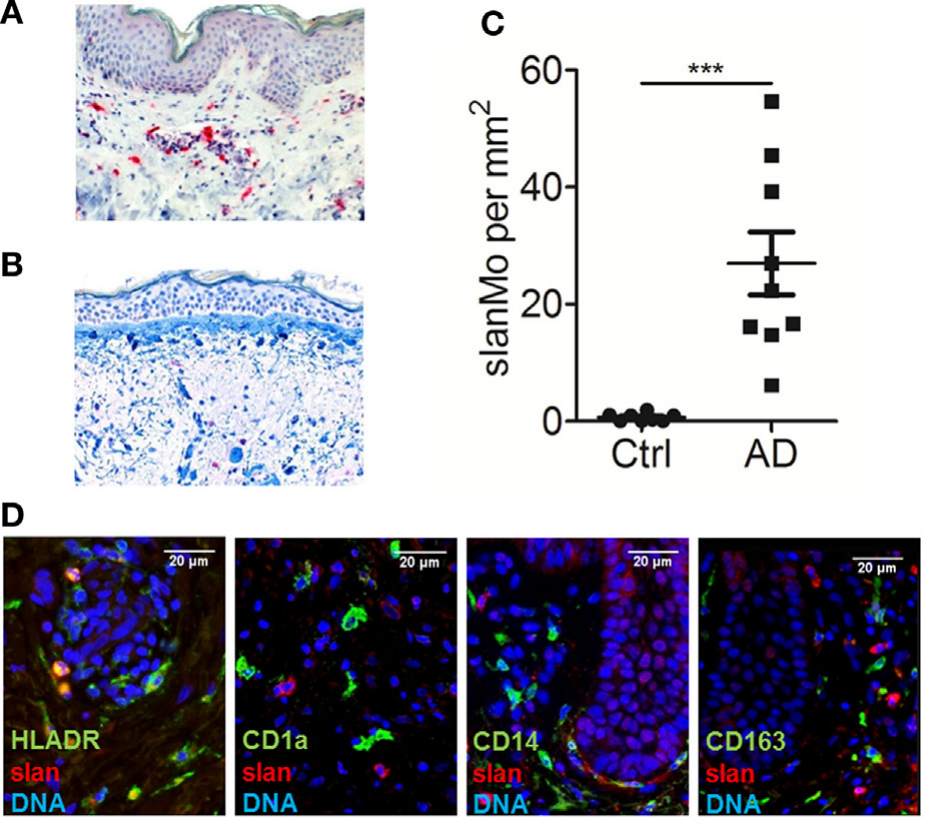
slan^+^ cells are a distinct population of dermal monocytes in atopic dermatitis. Immunohistochemical staining of 6-sulfo LacNAc-expressing monocytes (slanMo) in normal human skin **(A)** and skin samples from an atopic dermatitis patient **(B)**. The results of multiple samples are shown in **(C)**: healthy skin *n* = 10 and skin from atopic dermatitis patients *n* = 9. **(D)** Immunofluorescent stainings of slan (red) in combination with either HLA-DR, CD1a, CD14, or CD163 (green) was performed. Cell nuclei were stained with DAPI (blue). **(A,B,D)** Representative results of 10 donors that were analyzed. Statistical significance **(C)** was calculated using an unpaired *t*-test.

Flow cytometry results—slanMo frequency, phenotype, and cytokines production—were evaluated using CellQuest and WinMdi 2.8 software. Both Mean Fluorescence Intensity (MFI) and percentage of positive cells were measured and statistical differences were calculated using unpaired *t*-test, Mann–Whitney *U* test, or one-way ANOVA, where applicable. Two-tailed *p* values of less than 0.05 were considered statistically significant. In immunohistochemistry, DD2-positive cells (slanMo) were counted as number of cells per square millimeter dermis with use of ImageJ 1.38x software and differences between mean values of these cells were statistically compared. Immunofluorescent assay was evaluated as positive or negative double staining (Figure 1D).

## Results

### Distribution, Quantification, and Phenotype of slanMo in Atopic Dermatitis Skin Lesions

**Figure 2:**
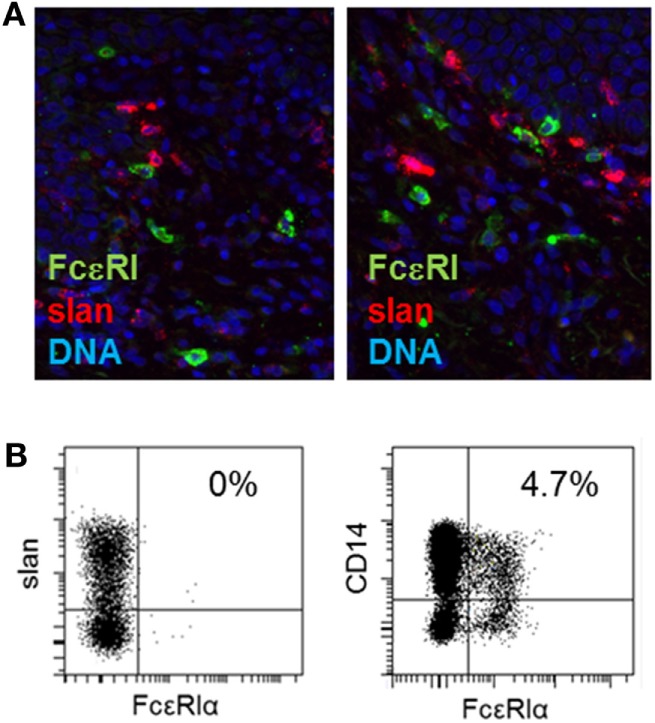
Expression of FcεRI by 6-sulfo LacNAc-expressing monocytes (slanMo) in skin and blood. **(A)** Immunofluorescent staining of slan (red) and FcεRI (green) in normal human skin (left) or skin from atopic dermatitis patients (right). Cell nuclei were stained with DAPI (blue). Representative results of 10 donors analyzed are shown. **(B)** Blood slanMo and CD14^+^ monocytes from patients with atopic dermatitis were analyzed for the expression of FcεRI by flow cytometry. A Representative result of 14 donors is shown.

In previous studies, slanMo were identified as a subset of TNF-α- and iNOS-expressing inflammatory dermal DCs in psoriasis, so called TIP-DCs (14, 25, 26). We hypothesized that this proinflammatory cell population may also contribute to the pathogenesis of AD. Skin biopsies of patients with AD and samples of healthy control skin were studied. slanMo were identified by immunohistochemistry using the mAb DD2. Few positively stained cells were found in the dermal compartment of healthy control skin samples (Figure 1A). In AD skin samples, slanMo were scattered throughout the upper dermis and clustered within the perivascular infiltrate (Figure 1B). The epidermis was devoid of slanMo. The number of dermal slanMo in patients was significantly higher than in healthy controls (Figure 1C). Extensive studies on psoriatic skin lesions demonstrated the specificity of the slan staining in tissue (13, 14). The phenotypical analysis of slanMo in lesional AD skin samples identified these cells as a HLA-DR^+^ cell population lacking expression of the Langerhans cell marker CD1a, the monocyte/DC marker CD14 and the macrophage marker CD163 (Figure 1C). FcεRI is expressed on different MPs in skin and blood of patients with AD (27, 28). It can facilitate uptake of IgE-complexed allergens and thereby enhance allergen-specific Th2 responses in the skin. Our studies on skin (Figure 2A, *n* = 10) and blood (Figure 2B, *n* = 10) did not reveal any evidence for expression of FcεRI by slanMo. In contrast, FcεRI expression was clearly detectable on skin MPs and among CD14^+^ blood classical monocytes (Figure 2). Combining results from previous studies and the data presented here, slanMo in skin and blood share the phenotype of a CD1a^−^, CD1c^−^, CD3^−^, CD14^−^, CD20^−^, CD163^−^ as well as FceRI-negative cell population with positive expression for CD11c, CD68, and HLA-DR (14).

### Phenotype of slanMo in Blood of Patients with AD

**Figure 3:**
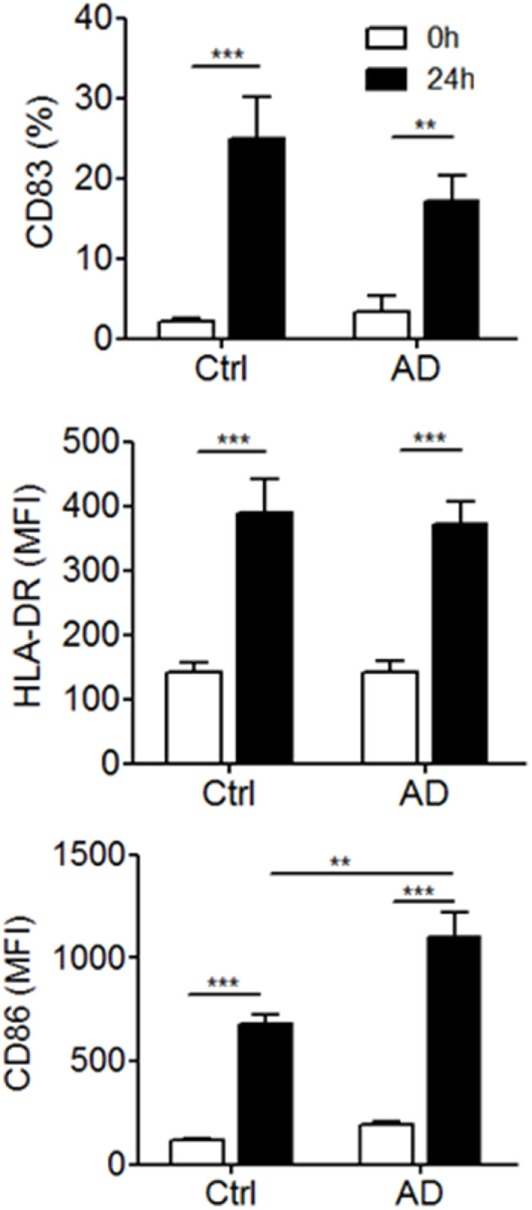
Expression of surface maturation markers by blood 6-sulfo LacNAc-expressing monocytes (slanMo) from healthy donors and atopic dermatitis patients. slanMo were stained within freshly isolated or 24-h cultured peripheral blood mononuclear cells and analyzed for the expression of HLA-DR, CD83, and CD86 by flow cytometry (Ctrl *n* = 10, AD *n* = 14). Statistical significance was calculated using a one-way ANOVA followed by Tukey’s posttest.

We next asked whether slanMo in blood of patients with AD differ in their phenotype and may show signs of activation or inhibition. In addition, we studied the capacity of slanMo from AD donors to undergo phenotypic maturation, a process spontaneously induced when these cells are cultured *in vitro* (29). Flow cytometric results for CD83 expression are given as percent positive cells, as it provides a better estimate of the distinct changes taking place during maturation, since slanMo are negative for CD83 after isolation but upregulate this marker during maturation. Instead, for HLA-DR and CD86, both of which are expressed on all slanMo, values of the MFI are provided. These studies on freshly prepared PBMCs revealed a similar expression of HLA-DR, CD83, and CD86 on slanMo of AD patients compared to healthy controls. The studied cell surface antigens are also sensitive markers of MP maturation (Figure 3; Figure S1 in Supplementary Material). slanMo from donors with AD revealed a strong upregulation of CD86 *in vitro*, which was significantly higher when compared to healthy control cells. The frequency of slanMo in blood of AD patients and control donors was not significantly different (Figure S2 in Supplementary Material).

### Cytokine Production of DCs and Monocytes in AD Patients

**Figure 4:**
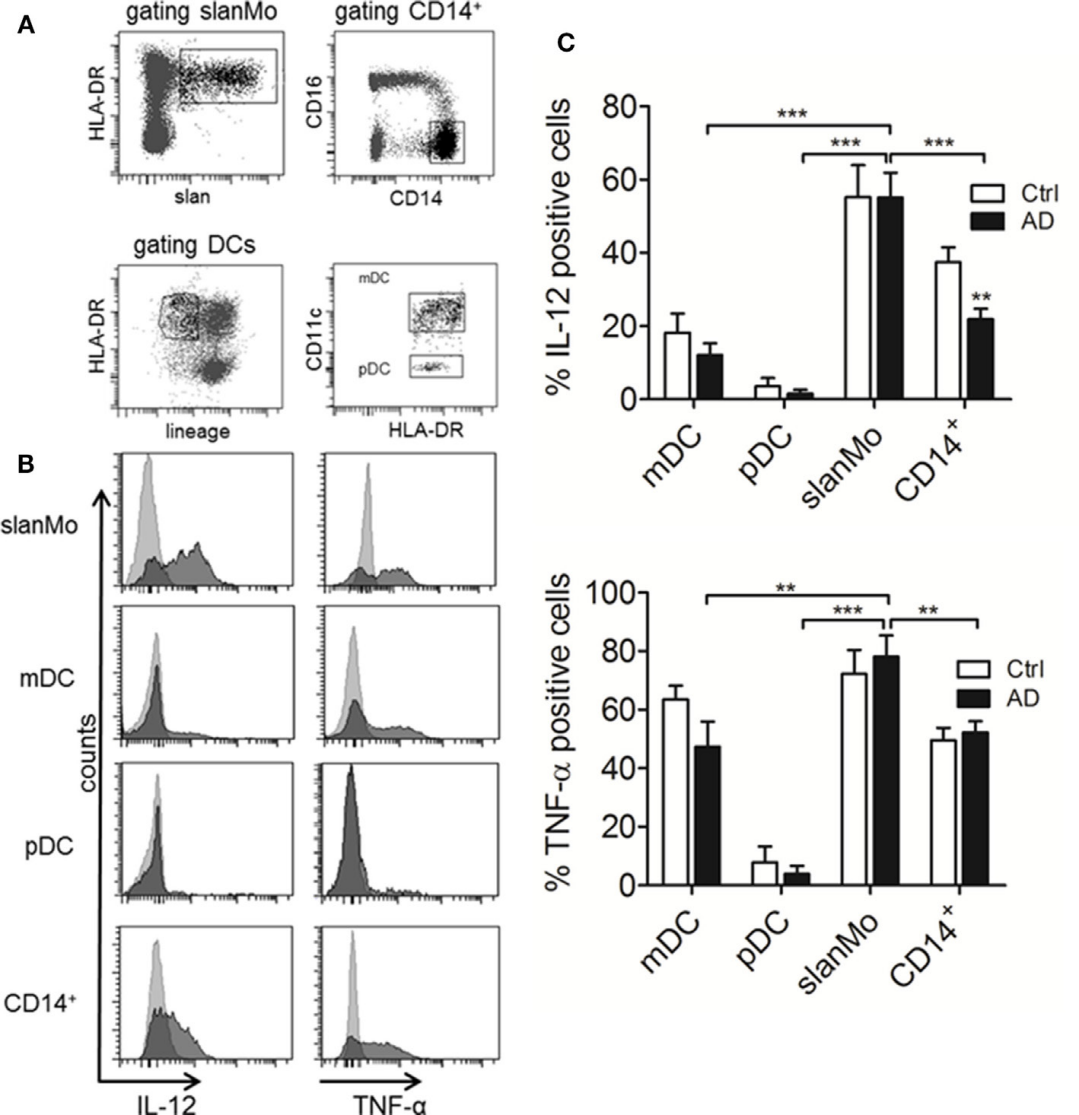
IL-12 and TNF-α production in different subtypes of dendritic cells (DCs) and monocyte subsets from healthy donors and atopic dermatitis patients. **(A)** Gating strategy for the different subsets analyzed in **(B,C)** is demonstrated. **(B)** Representative histograms for IL-12 and TNF-α production are shown. Results of multiple donors (Ctrl *n* = 11, AD *n* = 15) are depicted in **(C)**. Data are shown as mean + SEM. Statistical significance between Ctrl and AD within the different DC subsets was calculated using an unpaired *t*-test and a one-way ANOVA followed by Dunnett’s posttest was used to analyze differences in cytokine production between 6-sulfo LacNAc-expressing monocytes (slanMo) and the other subsets in AD.

IL-12p70 is produced when mature but not immature MPs are stimulated. For slanMo, it was shown that the threshold for maturation and IL-12p70 production is low and requires only 6 h of *in vitro* culture (29, 30). Therefore, PBMCs were cultured for 6 h before starting an 18-h stimulation with LPS, which was followed by studying the cytokine production at the single cell level by flow cytometry. Cell surface staining for slanMo, CD14^+^ monocytes, CD11c^+^ myeloid DCs, and CD11c^−^ pDCs was combined with intracytoplasmatic staining for cytokines (31). This method is suitable when using small blood samples and also circumvents the purification of multiple cell types possibly causing cell activation. Figure 4A gives an example of the gating strategy employed for identifying slanMo, CD14^+^ monocytes, myeloid DCs, and pDCs. Histograms (Figure 4B) give the staining results of LPS-stimulated cells for IL-12 and TNF-α of one representative donor with AD. Figure 4C summarizes the results of multiple donors tested. In these experiments, slanMo demonstrated a significantly higher capacity to produce IL-12p40/70 and TNF-α when compared to CD11c^+^ DCs (CD1c^+^ DCs and CD141^+^ DCs), pDCs, and monocytes. pDCs serve as an internal negative control as they do not express TLR4 and are not directly activated by the TLR4L LPS. Similarly, immature myeloid DCs are negative for TLR4 by RT-PCR (13). However, they upregulate TLR4 in culture, which in our studies is reflected by a robust LPS-induced production of TNF-α. For the parallel stimulation of myeloid DCs and slanMo—all expressing TLR7 and/or TLR8–R848 was added to the cultures. Again, slanMo produced significantly more IL-12 and TNF-α when compared with the different R848-stimulated DC subsets (Figures S3 and S4 in Supplementary Material).

Taken together in these experiments slanMo from patients with AD revealed a high production of IL-12p40/70 and TNF-α as did slanMo from healthy controls (Figure 4C). This is in difference to monocytes where we observed a significant reduction of the IL-12p40/70 production when cells from donors with AD were studied (Figure 4C).

### The Influence of Psychosocial Stress on the Frequency and TNF-α Production of slanMo

**Figure 5:**
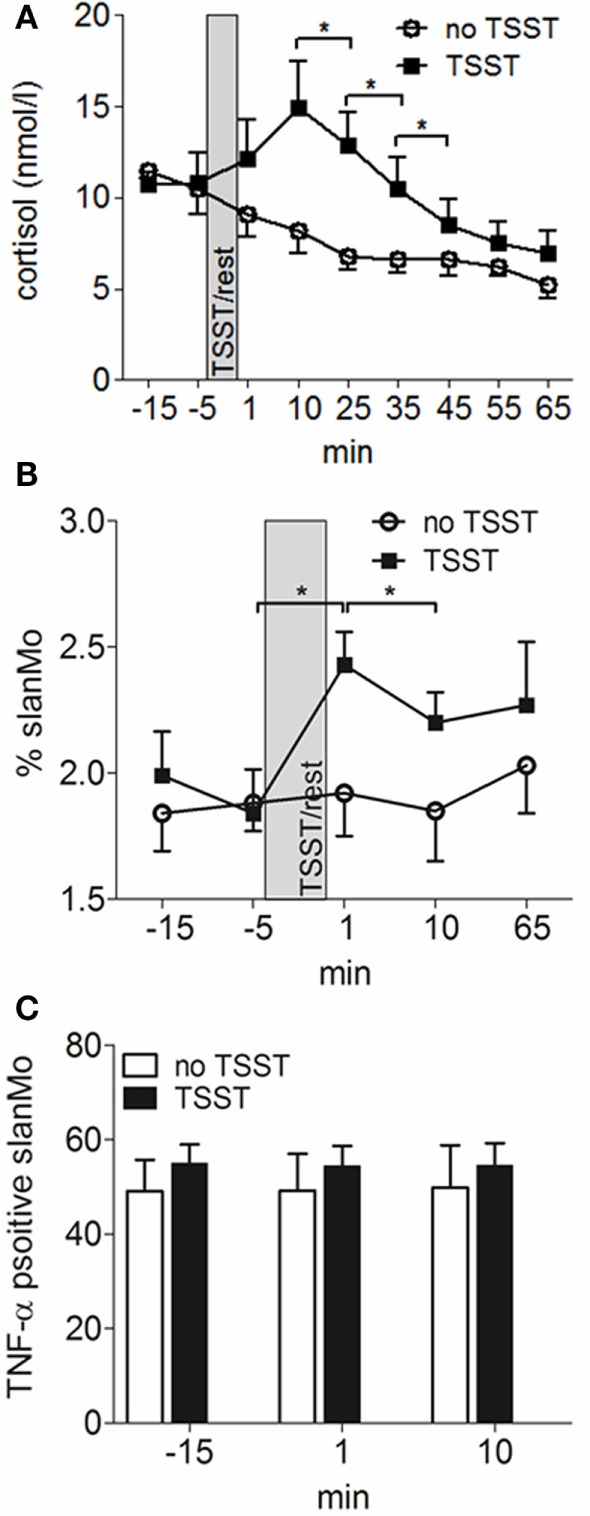
6-Sulfo LacNAc-expressing monocytes (slanMo) are mobilized by mental stress and retain their capacity to produce TNF-α. **(A)** Blood cortisol levels were measured before and after induction of mental stress [Tier Social Stress Test (TSST)] in atopic dermatitis patients or after a resting period (no TSST) (*n* = 12). Frequency of slanMo in peripheral blood mononuclear cells **(B)** and their TNF-α production **(C)** were determined by flow cytometry (*n* = 7). Data are shown as mean + SEM and statistical significance was calculated using a one-way ANOVA followed by Tukey’s posttest.

We next wanted to study how these cells are regulated by a common provoking factor of AD. We choose psychosocial stress as it is known to frequently cause acute eczematous flares of the disease (5). For these experiments, seven patients with AD underwent a standardized laboratory psychosocial stress test (Trier social stress test, TSST). The TSST is known to induce rapid changes in lymphocyte, monocyte, neutrophil and basophil numbers in blood and to cause an altered cytokine production (32). As already shown in previous studies, the acute mental stress caused a rapid and short-lived peak of cortisol levels (Figure 5A). The frequency of slanMo peaked directly after finishing the TSST and significantly declined within the following 10 min (Figure 5B). From previous studies it is known that following the TSST, TNF-α production of PBMCs stimulated with LPS may increase in healthy controls or decrease as shown in patients with chronic fatigue syndrome (33). To best monitor for immunomodulatory effects of experimental stress on slanMo, the obtained blood samples were directly propagated to isolate PBMCs and were directly stimulated with LPS for a period of only 6 h. PBMCs were collected 15 min before performing the TSST, 1 and 15 min following the TSST. When comparing the different time points for the TNF-α production of slanMo among LPS-stimulated PBMC, we did not find any significant differences. Therefore, these data clearly demonstrated that acute psychosocial stress mobilizes slanMo in patients with AD which, however, retained their high capacity to produce TNF-α.

## Discussion

With this study, we provide evidence for slanMo being a proinflammatory cell type in skin and blood of patients with AD.

Changes in the MP compartment in AD have been described previously. IDECs are a population of inflammatory DCs in AD having the phenotype of CD1a^+^ CD207^−^ FcɛRI^+^ cells (15). While IDECs are found in the epidermal and also in the dermal compartment of AD skin lesions, we identify slanMo as a dermal cell population. In difference to Langerhans cells, pDCs and IDECs, slanMo lacked expression of FcɛRI or CD1a and in contrast to classical monocytes, intermediate monocytes, and macrophages, slanMo were negative for CD14 and CD163. slanMo were found scattered in the upper dermis and within the perivascular lymphohistiocytic infiltrates. We identified FcɛRI^+^ CD14^+^ monocytes in blood of all atopic donors tested; however, slanMo in blood and dermis were negative for FcɛRI. In AD, expression of FcɛRI was initially described for CD1a^+^ Langerhans cells and was shown to facilitate internalization of IgE-bound allergens and stimulation of allergen-specific T cells (28, 34). Nevertheless, a mouse model with transgenic expression of human FcɛRI in DCs demonstrated a reduced severity of IgE-mediated food allergy and asthma, suggesting anti-inflammatory IgE/FcɛRI signals. Similarly, IgE/FcɛRI cross-linking inhibited production of proinflammatory cytokines of DCs (35). Therefore, it may be argued that lacking FcɛRI expression prevents slanMo from a negative feedback mechanism that would otherwise restrain their proinflammatory capacity.

6-Sulfo LacNAc-expressing monocytes were previously identified as IL-23-, TNF-α-, and iNOS-expressing inflammatory dermal DCs in psoriasis and thus are part of the population of inflammatory dermal DCs (TIP-DCs) in psoriasis (14). Due to their high IL-12, IL-23, TNF-α, and IL-1β production, slanMo are a proinflammatory population of cells programming T cells producing IFN-γ, IL-17, IL-22, but not IL-10 (14, 29). Therefore, slanMo are well equipped to support pathogenic T cell responses in AD where, in addition to Th2 cells also Th1, Th22 and also IL-17-producing T cells are present (36–38). For a direct side by side comparison of different MPs among PBMCs, we performed cell surface staining for CD11c^+^ DCs (conventional DCs, cDCs: CD1c^+^ DCs and CD141^+^ DCs), pDCs, slanMo, and CD14^+^ monocytes. slanMo from donors with AD produced higher amounts of IL-12/IL-23p40 compared to cDCs and CD14^+^ monocytes. Interestingly, CD14^+^ monocytes from donors with AD compared to healthy donors produced significantly less IL-12/IL-23p40. This finding is in accord with previous studies on monocytes as well as on monocyte-derived DCs, and CD1c^+^ DCs (39, 40). CD1c^+^ DCs are cDCs found in healthy skin, in psoriasis and at increased numbers in the dermis in AD (12). Studying LPS-stimulated cDCs we observed a minor but non-significant reduction in their IL-12 production. Notably, in patients with AD, LPS-induced IL-12 production by slanMo was not reduced. cDCs, pDCs, and slanMo differ in their expression of the LPS receptor TLR4, but all cells express either TLR7 or TLR8 (16). In a different set of experiments, we compared the response of these cells from patients with AD and healthy controls to the TLR7/8 ligand R848. These studies revealed similar findings for the production of IL-12, while with R848 stimulation a strong induction of TNF-α by pDCs became evident.

Different local factors shape the maturation and function of MPs in tissues and alter their IL-12 production. For slanMo, it was shown that IL-4 increases their IL-12 production and programming of Th1 cells, while reducing IL-23 production and programming of Th17 cells (41). Pollen-derived low-molecular weight factors inhibit the capacity of slanMo to induce Th1 responses (42). Similarly, histamine was shown to bind histamine 4 receptors on slanMo and thereby reducing their IL-12 production (43). So, it appears that slanMo, although having a strong inherent capacity to program Th1/Th17 responses, are controlled by different micromilieu factors.

Therapeutic antibody-directed inhibition of IL-4 and IL-13 receptors with dupilumab serve as a novel option to efficiently control disease in AD patients (44). In response to the successful therapy with dupilumab, inflammatory markers in lesional skin are downregulated (45). It is interesting to note that among the markers most strongly reduced in their expression by dupilumab are cytokines produced by slanMo (TNF-α, IL-23p19, and IL-12/23p40) themselves or by T cells induced by slanMo (IL-17, IFN-γ, and IL-22) (14, 45), thereby indicating that slanMo can support an immune response with relevance to AD.

In patients with AD, systemic inflammatory factors may also alter the phenotype and function of blood leukocytes. We demonstrated that slanMo from donors with AD mature and express higher cell surface levels of CD86 compared to mature slanMo from healthy donors. High expression of CD86 was shown to break immune tolerance by providing a strong T cell stimulatory capacity.

A hallmark of the clinical course of AD is the frequent flares of the disease. Among the different provoking factors of AD, mental stress is regarded as an important neuro-immunologic inducer of disease exacerbations (46). Having identified slanMo as a proinflammatory MP type in AD we asked for their frequency and function in the context of acute mental stress. We conducted the standardized Trier social stress test (TSST) with public speaking and mental arithmetic in seven patients with AD. Saliva cortisol levels temporarily increase as expected (47). Interestingly, the frequency of slanMo among PBMCs significantly increased 1 min after the TSST followed by a significant drop 10 min later. We also studied the immediate LPS-induced TNF-α production by intracellular cytokine staining in a 6-h stimulation assay. These studies demonstrated a high TNF-α production that was not altered studying slanMo mobilized by acute stress. Stress probably results in a catecholamine-mediated detachment of slanMo from the endothelium of the vascular walls in AD patients, comparable to findings published for healthy volunteers (48).

This study is the first to provide evidence for slanMo as being a relevant inflammatory cell type in AD. We identified slanMo as a frequent and phenotypically distinct subset of inflammatory dermal cells in AD skin lesions, demonstrated their high proinflammatory potential in blood of patients with AD, and documented their mobilization by acute psychosocial stress. These findings appear relevant for the pathogenesis of AD, as well as for future therapeutic strategies.

## Ethics Statement

This study was carried out in accordance with the recommendations of the ethics commission at medical faculties of Heidelberg (S033/2013, S03/2013, S305/2010, S306/2010) and Dresden (2602/2007). The protocol was approved by the ethics committee of Heidelberg and Dresden. All subjects gave written informed consent in accordance with the Declaration of Helsinki.

## Author Contributions

WB and SO performed experiments, analyzed data, and wrote the manuscript; FA, TD, and CA performed experiments and analyzed data; AB-K and MS designed research and critically revised the article; KS designed research, interpreted the data, and wrote the manuscript.

## Conflict of Interest Statement

The authors declare that the research was conducted in the absence of any commercial or financial relationships that could be construed as a potential conflict of interest.
