# Supplementary material for: Phenotype, Function, and Mobilization of 6-Sulfo LacNAc-Expressing Monocytes in Atopic Dermatitis
Source: Front Immunol. 2018 Jun 21;9:1352. doi: 10.3389/fimmu.2018.01352 (PMC6021776; doi:10.3389/fimmu.2018.01352)
Supplement: Supplementary file 1 [file data_sheet_1.docx]

Supplementary Material

Phenotype, function and mobilization of 6-sulfo LacNAc-expressing monocytes in atopic dermatitis

Wojciech Baran^1+^, Stephanie Oehrl^2+^, Fareed Ahmad^2^, Thomas Döbel^2^, Christina Alt^2^, Angelika Buske-Kirschbaum^3^, Marc Schmitz^4^, Knut Schäkel^2*^

^1^ Department of Dermatology, Venerology and Allergology, Wroclaw Medical University, Poland

^2^ Department of Dermatology, Medical Faculty, University of Heidelberg, Germany

^3^ Department of Biopsycology, Technical University of Dresden, Germany

^4^ Institute of Immunology, Medical Faculty, TU Dresden, Dresden, Germany; National Center for Tumor Diseases, University Hospital Carl Gustav Carus, TU Dresden, Germany; German Cancer Consortium (DKTK), Dresden, Germany; German Cancer Research Center (DKFZ), Heidelberg, Germany; Center for Regenerative Therapies Dresden (CRTD), Medical Faculty, TU Dresden, Dresden, Germany

^+^Both authors contributed equally

*** Correspondence:**Prof. Dr. Knut Schäkel, Department of Dermatology, Medical Faculty, University of Heidelberg

Im Neuenheimer Feld 440, 69120 Heidelberg, Germany; Tel. +496221568411; fax. +496221565406 email: knut.schaekel@med.uni-heidelberg.de

## 1. Supplementary Figures


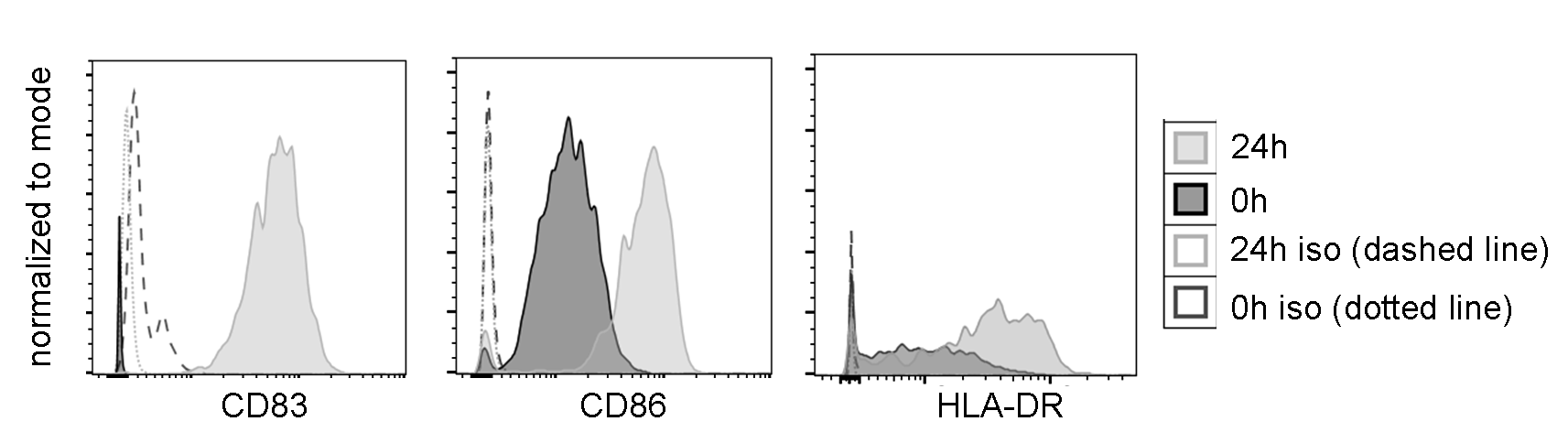


**Supplementary Figure 1. Representative histograms for the analysis of surface marker on slanMo.** slanMo were analyzed for the expression of CD83, CD86 and HLA-DR (or the respective isotype controls) directly after isolation (0h) and after 24h of culture.


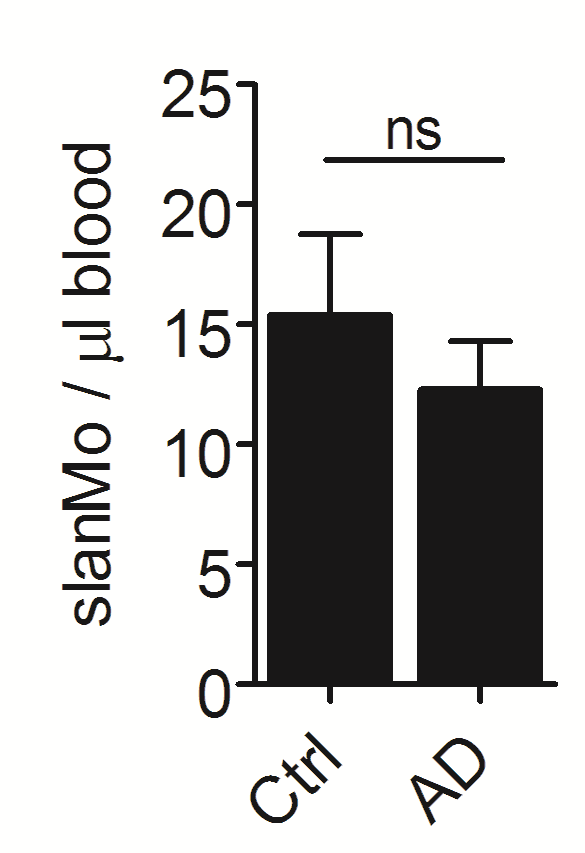


**Supplementary Figure 2. Frequency of slanMo in blood of atopic dermatitis patients and healthy controls.** Frequency of slanMo in whole blood was determined using counting beads in flow cytometric analysis (n=10). Statistical significance was tested using an unpaired t-test.


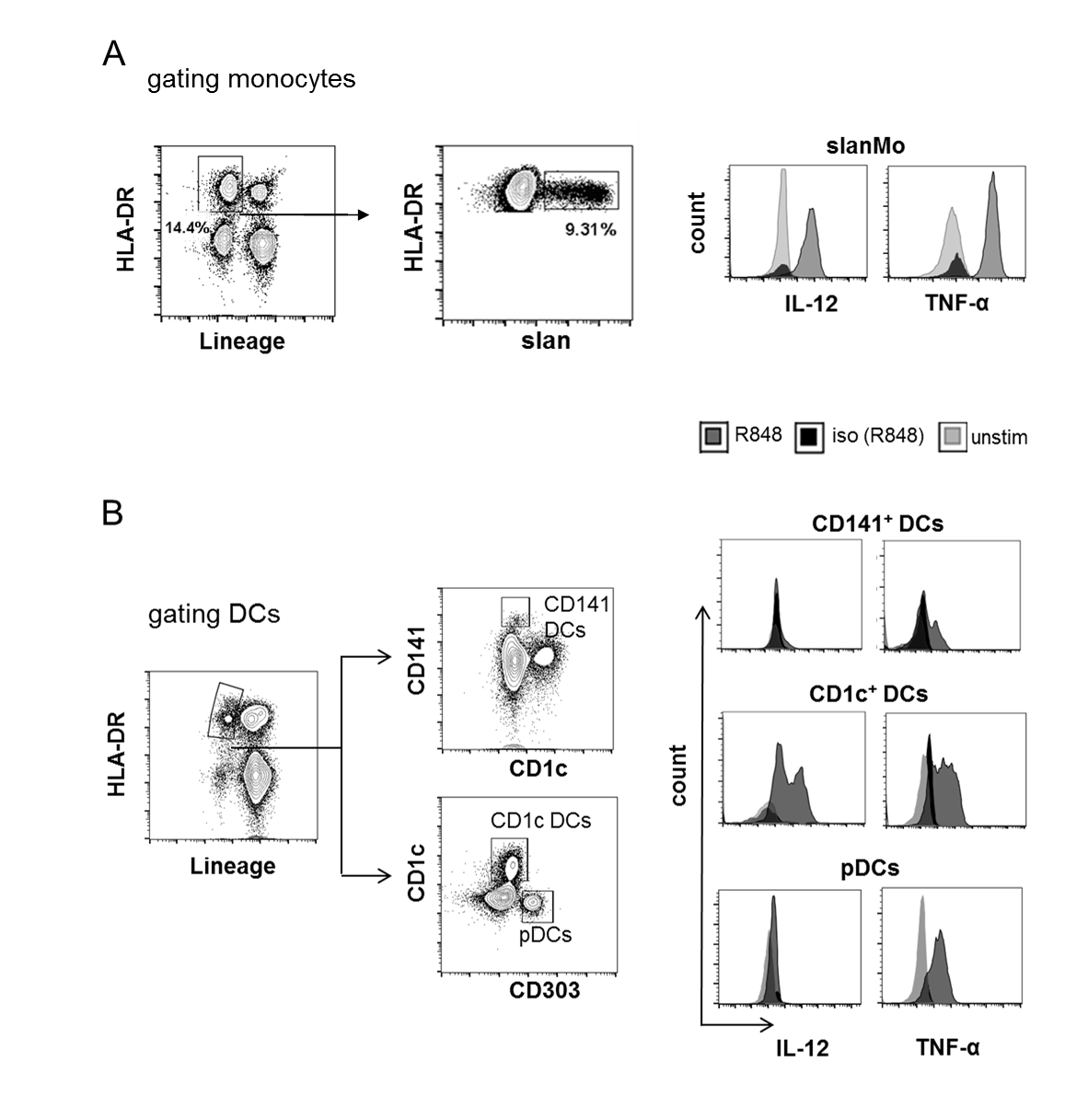


**Supplementary Figure 3. Gating strategy used to identify different monocyte and DC subsets.** In (a) the gating strategy for slanMo is demonstrated (lineage mix: CD3/CD19/CD56). The gating strategy for different DC subsets is shown in (b) (lineage mix: CD3/CD19/CD20/CD56/CD16/CD14). Representative histograms of the IL-12 and TNF-α production for the different cell populations are included.


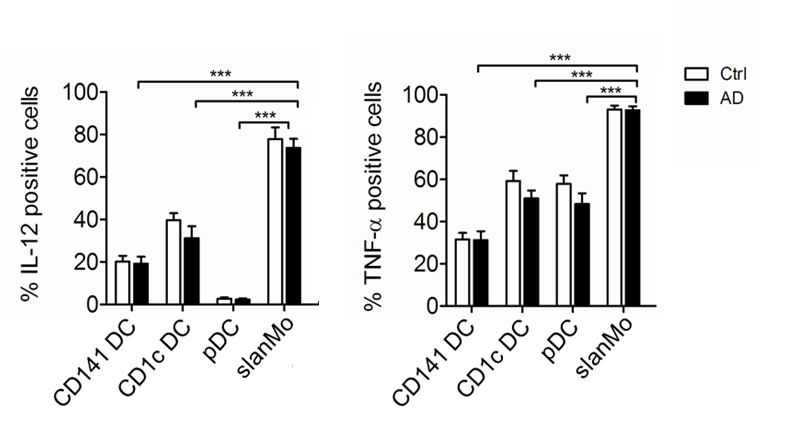


**Supplementary Figure 4.** **IL-12 and TNF-α production in different DCs and slanMo from healthy donors and atopic dermatitis patients after stimulation with R848.** Cells were stimulated with 5 µM of R848 for 18h after a 6h maturation period. Data are shown as mean+SEM (Ctrl: n=10, AD: n=14). Statistical significance was determined using one-way ANOVA followed by Dunnett’s posttest.

**2. Supplementary Material and Methods**

Cell preparation and flow cytometric analysis

Peripheral blood mononuclear cells (PBMCs) were prepared by Ficoll gradient centrifugation (Biochrom AG, Berlin, Germany) and resuspended in RPMI medium supplemented with 2 mM L-glutamine, 1% non-essential amino acids, 100 U/mL penicillin, 100 µg/mL streptomycin and 10% heat-inactivated pooled human AB serum (CC Pro, Oberdorla, Germany) and stimulated at 6 hours of culture with 5 µM R848 (Invivogen, Toulouse, France) ) supplemented with 1 µg/ml brefeldin A (Sigma-Aldrich) to inhibit cytokine secretion and cultured for a total of 24 hours (37°C at 5% CO2). For intracytoplasmic cytokine staining PBMCs first underwent cell surface staining to identify slanMo, CD1c^+^ DCs, CD141^+^ DCs and pDCs. Cells were fixed in fresh ice-cold 4% paraformaldehyde, and permeabilized using 0.1% saponin in PBS containing 1% FCS. For intracellular cytokine staining an anti-TNF-α mAb (clone MAb11), an anti-IL-12/23p40 mAb (clone 11.5, both BD) and respective isotype controls were used. The frequency of slanMo in blood of healthy controls and AD patients was determined by flow cytometry using counting beads.
